# Memory Matters: Education About Cognition in Two Naturally Occurring Retirement Communities

**DOI:** 10.1093/geroni/igaa057.942

**Published:** 2020-12-16

**Authors:** Gregory Hinrichsen, Joyce Fogel, Anne Foerg, Rosanne Leipzig

**Affiliations:** 1 Icahn School of Medicine at Mount Sinai, New York, New York, United States; 2 JASA, New York, New York, United States

## Abstract

“Memory Matters” is a program for older residents and staff of two New York City naturally occurring retirement communities (NORC). The program is in its third year of implementation with the broader goals of enhancing resident knowledge of normal cognitive changes and cognitive impairment including risk factors for dementia. The emphasis is on engaging residents to improve brain health. With staff, goals are to enhance their capacity to understand, identify, refer, and support residents with cognitive impairment. This is done with case conferences, consultation, lectures, and workshops. For residents, the program provides lectures, interactive workshops, films, as well as periodic “Ask the Doc” individual meetings where residents can discuss cognitive and related concerns. A geriatrician and geropsychologist conduct these activities with invited guest speakers including geriatric fellows. Yearly, approximately 180 unique individuals are served in this program. Resident program satisfaction surveys were favorable. For example, 80% of residents replied “a lot” to the question, “Did you find this presentation interesting?”, in the past six months of programming. Building on these efforts, we conducted a formal survey with 249 resident participants of one NORC that found: 15% have dementia/experience memory loss; 12% live with someone with dementia/ memory issues; 16% identify as a dementia caregiver; and 75% are concerned about developing dementia. We then hosted a community-wide forum to discuss survey results. Our experience has underscored the need for expansion of our efforts. Future plans include creating a “dementia friendly” NORC. Funded by the UJA-Federation of New York.

